# Modeling and Experimental Results of Selected Lightweight Complex Concentrated Alloys, before and after Heat Treatment

**DOI:** 10.3390/ma13194330

**Published:** 2020-09-29

**Authors:** Dumitru Mitrica, Ioana Cristina Badea, Mihai Tudor Olaru, Beatrice Adriana Serban, Denisa Vonica, Marian Burada, Victor Geanta, Adrian Nicolae Rotariu, Florentin Stoiciu, Viorel Badilita, Lidia Licu

**Affiliations:** 1 National R&D Institute for Nonferrous and Rare Metals—IMNR, 102 Biruintei Blvd, Pantelimon, 077145 Ilfov, Romania; cristina.banica@imnr.ro (I.C.B.); beatrice.carlan@imnr.ro (B.A.S.); denisav22@gmail.com (D.V.); mburada@imnr.ro (M.B.); fstoiciu@imnr.ro (F.S.); vbadilita@imnr.ro (V.B.); lidia.licu@imnr.ro (L.L.); 2 Faculty of Materials Science and Engineering, University POLITEHNICA of Bucharest, 313 Splaiul Independenței, 6 District, 060042 Bucharest, Romania; victorgeanta@yahoo.com; 3 Military Technical Academy, 39-49 George Coşbuc Avenue, 050141 Bucharest, Romania; arotariu99@yahoo.com

**Keywords:** complex concentrated alloys, low weight, modeling, heat treatment, characterization

## Abstract

Lightweight complex concentrated alloys (LWCCA), composed of elements with low density, have become a great area of interest due to the high demand in a large number of applications. Previous research on LWCCAs was focused on high entropy multicomponent alloy systems that provide low density and high capability of solid solution formation. Present research introduces two alloy systems (Al-Cu-Si-Zn-Mg and Al-Mn-Zn-Mg-Si) that contain readily available and inexpensive starting materials and have potential for solid solution formation structures. For the selection of appropriate compositions, authors applied semi-empirical criteria and optimization software. Specialized modeling software (MatCalc) was used to determine probable alloy structures by CALPHAD, non-equilibrium solidification and kinetic simulations. The selected alloys were prepared in an induction furnace. Specimens were heat treated to provide stable structures. Physicochemical, microstructural, and mechanical characterization was performed for the selected alloy compositions. Modeling and experimental results indicated solid solution-based structures in the as-cast and heat-treated samples. Several intermetallic phases were present at higher concentrations than in the conventional alloys. Alloys presented a brittle structure with compression strength of 486–618 MPa and hardness of 268–283 HV. The potential for uniform intermetallic phase distribution in the selected alloys makes them good candidates for applications were low weight and high resistance is required.

## 1. Introduction

Complex concentrated alloys (CCA) represent a new group of materials, with complex microstructures, comprising of a large number of elements [1]. CCAs extend the established limits of the high entropy alloys (HEAs) concept by allowing a smaller number of elements in the composition and in various proportions, that can deliver mixed structures containing solid solution and well dispersed intermetallic phases. CCAs generally have higher configurational entropy than conventional alloys and are capable of obtaining complex, disordered compositionally complex solid solution structures. CCAs are distinct from conventional alloys in that they are not based on a single major element and rather include several main elements, which allow a high degree of freedom in alloy compositions and properties. Their properties, such as high temperature resistance, corrosion resistance, workability, and low density, offer a remarkable advantage in the use of CCAs for various extreme conditions applications [2,3].

While no attention was directed to light density CCAs yet, the development of the research in the field of materials science and engineering has focused on the progress of lightweight high-entropy alloys (LWHEA), with superior mechanical and functional properties. Due to their similar design philosophy HEAs are included in the CCA group of alloys, thus a presentation of the most appropriate efforts in developing LWHEA is relevant also to the research of light density complex concentrated alloys.

Several studies in achieving lower density HEA were performed by several authors. Senkov et al. [4,5] studied the microstructure and mechanical properties of low density (up to 6.57 g/cm^3^) refractory HEAs. NbTiVZr, NbTiV_2_Zr, CrNbTiZr, and CrNbTiVZr alloys were developed by replacing Mo, W, and Ta with Ti, Zr, V, and Cr. Results showed comparable mechanical properties at high temperatures with Inconel 718 and Haynes 230, and CrNbTiVZr largely exceeded the superalloys characteristics (1298 MPa at room temperature and 259 MPa at 1000 °C). Another method for lowering density and improving corrosion resistance in refractory alloys is the addition of Al [6]. Among several compositions, two alloys (AlMo_0.5_NbTa_0.5_TiZr and AlNb_1.5_Ta_0.5_Ti_1.5_Zr_0.5_) have distinguished showing low density (7.4 g/cm^3^ and 6.9 g/cm^3^, respectively) and remarkable strength (745 MPa and 403 MPa, respectively) at high temperatures (1000 °C), exceeding IN718 and Mar-M247 performance.

A concentrated effort in the development of low density HEAs involved several research teams. In order to improve the mechanical properties of magnesium alloys Li et al. [7] prepared Mg_x_(MnAlZnCu)_100-x_ with variable Mg concentration. The alloys were characterized by high compressive strength (500–400 MPa) and hardness (431–178 HV). The as-cast alloy formed a stable Al-Mn icosahedral quasicrystal phase which is usually met in the alloys obtained by the rapid solidification process.

A high entropy alloy with very low-density (2.67 g/cm^−3^) was prepared by Youssef et al. [8] by mechanical alloying of elemental powders. The Al_20_Li_20_Mg_10_Sc_20_Ti_30_ alloy presented a single-phase structure, with a nanocrystalline grain size of 12 nm and a mechanical hardness of 5.9 GPa.

A different strategy was applied by Feng et al. [9] in selecting LWHEA by the gradual additions of Al and Ti to a core group of transitional elements, in equal proportion (CrFeMn). In order to decrease the probability for the formation of intermetallic phases, authors limited the Ti molar ratio to 0.25 and 1. The criteria calculations for solid solution formation did not satisfy the experimental results, which were well defined by the CALPHAD findings. All the alloys contained intermetallic based phases, but with dominant BCC structure in Al_1.0_CrFeMnTi_0.25_ and Al_2.0_CrFeMnTi_0.25_ alloys.

Al and Si additions to CuFeMnNi alloy, as well as the influence of cooling rates on the phase constitution of the alloy, were investigated by Kushnerov and Bashev in [10]. Most of the alloy compositions formed single phase FCC structures in the as-cast state. The increase in the cooling rate through rapid solidification negatively influenced the mechanical properties of the alloy.

Tseng et al. [11] developed a LWHEA containing Be, with a predominant solid solution structure and low level of intermetallic phases, obtained by arc melting. The A_l20_Be_20_Fe_10_Si_15_Ti_35_ (atomic percentage composition) alloy delivered unique mechanical properties and oxidation resistance which makes them appropriate for applications in the transportation industry.

Sanchez et al. [12] studied three lightweight high entropy alloys Al_40_Cu_15_Cr_15_Fe_15_Si_15_, Al_65_Cu_5_Cr_5_Si_15_Mn_5_Ti_5_ and Al_60_Cu_10_Fe_10_Cr_5_Mn_5_Ni_5_Mg_5_ (atomic percentage composition), which were previously designed by CALPHAD modeling. The alloys were produced by large scale vacuum die casting. Even if the cooling rate of the alloys was very low, the number of phases predicted by Thermo-Calc software was larger than those found in the experimental samples. The obtained alloys presented a high hardness to density ratio, due to the presence of complex precipitates in the as-cast structure.

Most of the literature reports in the field of LWHEA were discussing the formation of single-phase or predominant solid solution structures, in compositions containing elements with low density and with capability to form solid solutions. Other elements with considerable higher density were also included to avoid a high proportion of intermetallic phases. Nevertheless, light density metals are generally very reactive and easily form unwanted oxides and/or intermetallic compounds in the resulted alloy structures. In this respect, there are few metals that can be successfully used for the design of LWHEA. Al, Si, Mg, Ti, Li, Sc, and Be are few of more accessible elements to include in alloys that could be destinated to industrial processing. Previous efforts in alloying exclusively light-density elements were limited to the obtaining by nonequilibrium methods such as mechanical alloying [13], which would increase substantially the cost of the final product. Most of the LWHEA prepared by furnace melting were limited to a composition containing Al, Si, Mg, Ti, or Be, with the addition of higher density elements, such as Cu, Fe, Mn, Cr, and Ni, which determined an increase in alloy’s density. However, the obtained alloys presented improved properties, exceeding the conventional alloys characteristics and presented a promising approach in the continuously demanding field of materials design.

In order to determine the most relevant HEA compositions, a set of theoretical and empirical criteria was developed by several authors [14,15,16,17,18,19,20,21]. The formation of predominant solid solution phases and moreover the obtaining of single phase HEAs, based on specific elemental combinations, were studied by a semiempirical approach, where the theoretical criteria were combined with experimental findings. Hume-Rothery rule applied to the high entropy alloys showed that for atomic size difference (δ) less than 6.6 [15,16] and Allen electronegativity difference (Δχ_Allen_) between 3 and 6 [17,18], solid solution structures will be preferentially formed in as-cast alloys. The valence electron concentration (VEC) determined the type of solid solution structure which is FCC for VEC less than 6.87, a mix of FCC and BCC structures found if 6.87 < VEC < 8, and predominant BCC structures should appear if VEC is higher than 8 [19]. Thermodynamic parameters like enthalpy of mixing (−11.6 kJ/mol < ΔHmix < 3.2 kJ/mol), entropy of mixing (11 J/mol·K < ΔSmix < 19.5 J/mol·K) or ratio between them (1.1 < Ω), are other types of criteria, which are also used to determine solid solution formation in high entropy alloys [15,20]. A separate criterion based on the influence of the atomic size difference on the mixing entropy of the system has been determined and named geometric parameter Λ, which shows that predominant solid solutions are formed for Λ *<* 0.24, while single phase solid solution forms when Λ *>* 0.96 [21]. The mentioned criteria are not exhaustive and are not meant to be effective on any CCA system, as was proven before in referenced papers.

CALPHAD is another method for selecting HEA compositions, that implies complex computer modeling, based on thermodynamic calculations and empirical data. The method was proposed, performed, and recommended by several authors [22,23,24,25,26]. CALPHAD method paired with established semi-empirical criteria and diffusion modeling is able to offer a sustainable approach in selecting most viable compositions. The calculation results can be than compared to the experimental findings.

The present study investigates several lightweight complex concentrated alloys (LWCCA) composed of readily available elements, that are widely used in conventional alloys, based on Al-Cu-Si-Zn-Mg and Al-Mn-Zn-Mg-Si systems. Our purpose was to design a proper alloy with inexpensive lightweight elements, who can be obtained with ease by traditional manufacturing processes. Alloy design tools were used to predict structural behavior before and after the heat treatment process and experimental trials were performed for the selected alloys.

## 2. Materials and Methods

In order to reach the desired physical and mechanical properties, it is very important to find a suitable composition of the alloy. The properties of CCAs are specifically influenced by the constitutive elements. A vast majority of lightweight high-entropy alloys contain elements like Al, Si, Mg, Ti, and B in order to reduce alloy density. In order to avoid alloy brittleness due to poor machinability, elements like Cu, Zn, and Mn were selected as most appropriate. Although, these elements are not known as having low density, they are able to form stable solid solutions with lighter elements. Thus, Mn favour formation of complex solid solutions and Cu induces high machinability of the material by increasing deformability.

Selection of most appropriate compositions was performed by the optimization of semi-empirical criteria. The semi-empirical criteria are defined by the following relations:

- The entropy of mixing (ΔS_mix_) is calculated using Boltzmann’s equation
  (1) $${\Delta S}_{\mathrm{mix}}=-R\times \sum c_{i}\times {\mathrm{lnc}}_{i}$$
  where R is the gas constant (8.314 J/mol·K) and c_i_ is the molar fraction for element i,
- The enthalpy of mixing (ΔH_mix_) is calculated with a formula derived from the Miedema macroscopic model [27]
  (2) $$\Delta H_{\mathrm{mix}}=\sum 4c_{i}c_{j}\times \Delta H_{\mathrm{ij}}$$
  where ΔH_ij_ is the binary enthalpy of mixing for elements i and j
- The atomic size difference (δ) was defined by [14]
  (3) $$\delta =100\times \sqrt{\sum c_{i}\times {\left(1-\frac{r_{i}}{\bar{r}}\right)}^{2}}$$
  where r_i_ is the atomic radius of element i, $\bar{r}$ is the average atomic radius.
- The derived parameter Ω, which includes the influence of both ΔS_mix_ and ΔH_mix_ [15], was calculated with
  (4) $$\Omega =T_{m}\Delta S_{mix}/\left|\Delta H_{mix}\right|$$
  where *T_m_* is the melting temperature calculated with $T_{m}=\sum c_{i}\times T_{mi}$, and *T_mi_* is the melting temperature of element *i*.
- The formula for electronegativity difference after Allen (Δχ) was deduced as follows [17]
  (5) $$\Delta \chi =100\times \sqrt{\sum c_{i}\times {\left(1-\frac{\chi_{i}}{\bar{\chi }}\right)}^{2}}$$
  where χ_i_ is the Pauling electronegativity of element i and $\bar{\chi }$ is the average electronegativity.
- The valence electron concentration (VEC) is used for determining the type of solid solution formed in the alloy. VEC is calculated with an equation based on element concentration and individual VEC [19]
  (6) $$VEC=\sum c_{i}\times VEC_{i}$$
  where *VEC_i_* is the valence electron concentration of element *i*.
- The geometrical parameter (Λ) that defines the ration between mixing entropy and atomic size difference of the alloy [21]
  (7) $$\Lambda ={\Delta S}_{\mathrm{mix}}/\delta^{2}$$

The properties for the elements that enter the calculations were acquired from valuable sources, suggested by the authors that originally developed the criteria, such as: atomic radius [28], Allen electronegativity [29,30], melting temperatures [31], and pair mixing enthalpy [32].

The optimization of the alloy compositions was performed by means of the multi-objective optimization module from MATLAB software (version 6.02, MatCalc Engineering GmbH, Vienna, Austria).

MatCalc Pro edition, version 6.02, was used for multi-component phase equilibrium and thermodynamics by CALPHAD method, as well as non-equilibrium solidification and multi-phase precipitation kinetics.

The optimal alloy compositions were prepared in an induction furnace type Linn MFG-30 (Linn High Therm GmbH, Eschenfelden, Germany) with inert atmosphere and cast in a copper mould. Technical purity raw materials of Al, Cu, Si, Zn, Mg, and Mn were used in the experimental trials. A 350 g charge for each alloy was placed in an alumina-based crucible. The alloys were melted in the induction furnace and cast in a copper mould, under protective atmosphere. The resulted as-cast alloys were annealed in an electrical furnace, LHT 04/17 Nabertherm GMBH (Lilienthal, Germany), under protective atmosphere (Ar). The heat treatment stage was conducted at 400 °C for 20 h, with slow cooling rate of 3 °C/min. Samples were taken before and after the heat treatment process for chemical, structural, and mechanical analyses.

The chemical composition of the alloys was determined by inductively coupled plasma spectrometry (ICP-OES) using an Agilent 725 spectrometer (Santa Clara, CA, USA). Samples from various places of the alloy ingot were investigated. Optical microscopy investigation was performed with a Zeiss Axio Scope A1m Imager microscope (Jena, Germany) with bright field, dark field, DIC and polarization capabilities, and high contrast EC Epiplan 109/509/1009 lenses. Image analyzer software ImageJ vers. 1.53e, developed by W. Rasband, U. S. National Institutes of Health, Bethesda, MD, USA, was used to count porosity and shrinkage in the specimens. Samples were previously etched with a Keller type solution (prepared on-site) to enhance the visibility of the grains and the grain boundaries. Scanning electron microscopy (SEM) was performed with a FEI Quanta 3D FEG microscope (FEI Europe B.V., Eindhoven, Netherlands), operating at 20–30 kV, equipped with an energy dispersive X-ray spectrometer (EDS). The phase configuration was analyzed by X-ray diffractometry (XRD) (FEI Europe B.V., Eindhoven, Netherlands). Data acquisition was performed on BRUKER D8 ADVANCE diffractometer (Bruker Corporation, Billerica-MA, USA), using DIFFRAC^plus^ XRD Commender (Bruker AXS) software (version 2018, Bruker Corporation, Billerica-MA, USA), Bragg-Brentano diffraction method, Θ - Θ coupled in vertical configuration, with the following parameters: CuKα radiation, 2Θ Region: 20–1240, 2Θ Step: 0.020, Time/step: 8.7 s/step, rotation speed 15 rot/min, Cukβ radiation was removed with SOL X detector (Bruker Corporation, Billerica, MA, USA). The resulting data was processed using Bruker^®^ Diffracplus EVA Release 2018 software to search the database ICDD^®^ Powder Diffraction File (PDF4+, 2019 edition) and the full pattern matching (FPM) module of the same software package. The semi-quantitative evaluation was performed by the RIR method (reference intensity ratio) of the identified phase concentrations.

Compression strength for both alloys was determined with a LBG testing machine, model TC-100 (LBG testing equipment srl, Azzanos, Paolo, Italy), with max load of 100 kN. The testing samples were alloy bars with 6 mm in diameter and 6 mm length. The compression speed was 6 mm/min.

Vickers microhardness of the samples was measured at room temperature using microindenter attachment (Anton Paar MHT10, Anton Paar GmbH, GRAZ, Austria), with an applied load of 2 N and slope of 0.6 N/s. Ten measurements were made for each sample to determine the average values.

## 3. Results

### 3.1. Criteria Calculation

The criteria calculations were performed for the Al-Cu-Si-Zn-Mg and Al-Mn-Zn-Mg-Si systems (Table 1) with the variation of each element composition in a relevant interval, appropriate to the high entropy concept. Each element from the alloy composition was varied separately from 0.5 to 2.5 molar concentrations, according to the high entropy alloy conventions (1 is the value for equal composition).

**Figure 1:**
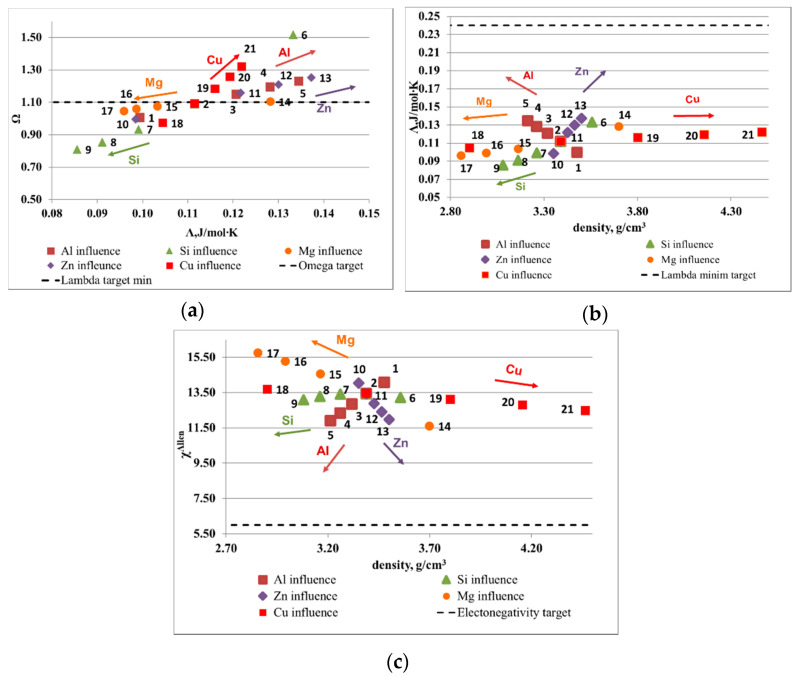
Elemental influence over the criteria values for Al-Cu-Si-Zn-Mg system: (**a**) Λ versus Ω, (**b**) Λ versus density, and (**c**) ∆χ_Allen_ versus density.

**Figure 2:**
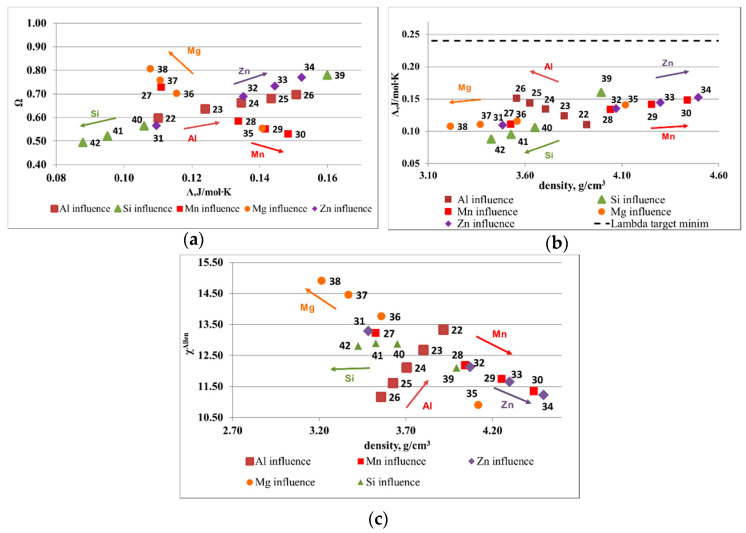
Elemental influence over the criteria values for Al-Mn-Zn-Mg-Si system: (**a**) Λ versus Ω, (**b**) Λ versus density, and (**c**) ∆χ_Allen_ versus density.

The influence of element composition on the main parameters is illustrated in Figure 1 and Figure 2. The optimal ranges for solid solution formation, as presented in the introduction section, are: Ω > 1.1, ∆χ_Allen_ < 6% and Λ > 24 J/mol·K. The atomic size mismatch was not represented as is included in the Λ parameter. The range limit is shown on diagrams with a dotted line. The results show that, for a Al-Cu-Si-Zn-Mg system, Mg and Si are decreasing the probability for solid solution formation, while Al, Cu, and Zn have a positive effect. However, Cu and Zn are rather heavy elements and increase the alloy density considerably, which has to be taken into account in final alloy selection. The Al-Mn-Zn-Mg-Si system capability to form solid solutions is strongly improved by the increase of Al concentration. Mn and Zn have also a positive influence on the alloy structure, but less important than Al. Another contradiction is shown here between Ω and Λ parameter, for the Mn and Mg influence. As expected, Si is not a good solid solution former, similar to the effect on Al-Cu-Si-Zn-Mg system.

However, the results showing the influence of each element to the formation of solid solution structures are qualitative in nature and do not show specific alloy compositions that can be further studied for practical use. The influence of the composition elements on the structural behavior is important in material design for determining compositional areas of interest that can be attractive for different applications. Those areas can then be studied further for the development of optimal compositions with targeted properties.

Taking data acquired in the present research work into consideration, a general approach for the selection of optimal compositions with high probability in the formation of solid solution structures can be performed by the means of special algorithms of global multi-objective optimization process.

In order to select most appropriate compositions for both systems, the equations defined by the established criteria entered a multi-objective optimization process. There are several methods that can be applied to present case scenario for multi-objective optimization. The Pareto front method was selected for increased accuracy. The optimization problem was set for finding the minimum value for the electronegativity difference (Equation (5)) and alloy density (Equation (8)), and the maximum value for the derived parameter Ω (Equation (4)) and geometrical parameter Λ (Equation (7)). The density of the alloy was calculated with formula

(8) $$\frac{1}{\rho }=\sum_{i=1}^{n}\frac{w_{i}}{\rho_{i}\times 100}$$

where *ρ* is the alloy density, *ρ_i_* is the component metal density, and *w_i_* is the concentration in weight percent (wt.%) of the component metal *i*.

The problem setup contained the constraints for optimal values, which correspond to the limits for each parameter presented earlier in the introduction section: *Λ* > 0.24, Ω > 1.1 and Δχ_Allen_ < 6. The genetic algorithm solver was used with following options: double vector population, feasible population for creation function, adaptive feasibility for the mutation function, and single point crossover function.

**Figure 3:**
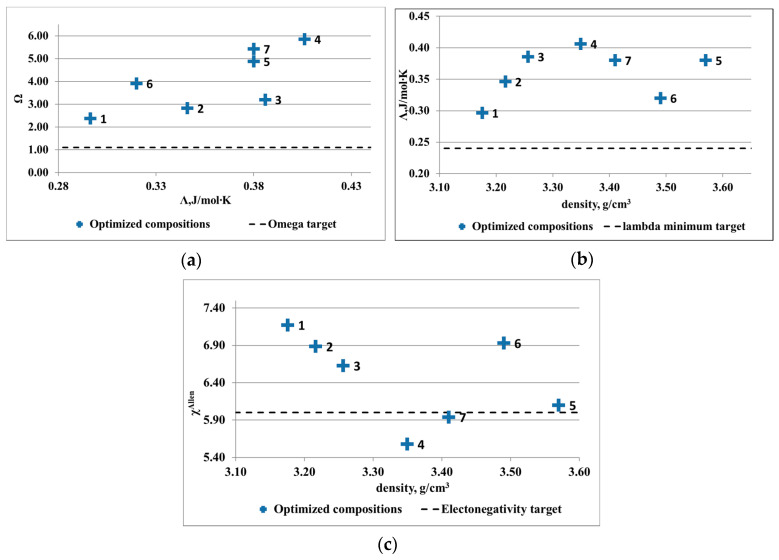
Results of criteria multiobjective optimization for Al-Cu-Si-Zn-Mg system: (**a**) Λ versus Ω, (**b**) Λ versus density, and (**c**) ∆χ_Allen_ versus density.

**Figure 4:**
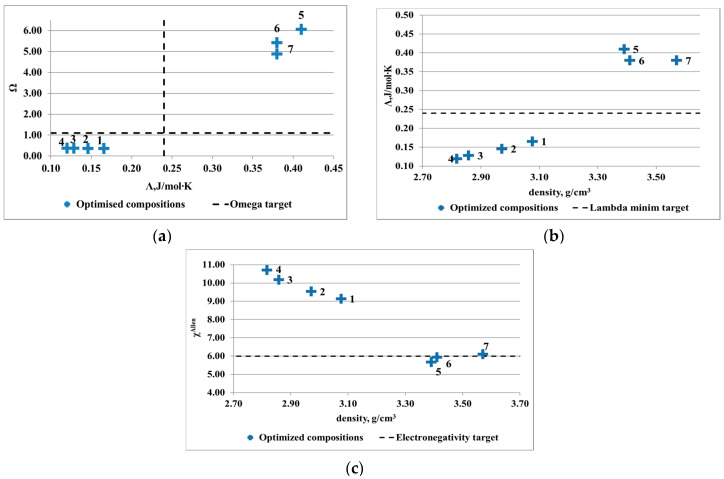
Results of criteria multiobjective optimization for Al-Mn-Zn-Mg-Si system: (**a**) Λ versus Ω, (**b**) Λ versus density, and (**c**) ∆χ_Allen_ versus density.

Several optimized compositions were determined (Table 2 and Table 3) and were illustrated in Figure 3 and Figure 4. For the Al-Cu-Si-Zn-Mg system, several alloy compositions are complying with the solid solution formation criteria. Even if the electronegativity criterion is respected only by two compositions, the rest of the alloys are not far away from the solid solution formation limit. The lowest density was shown by the Al_3.4_Cu_0.5_Si_0.2_Zn_0.5_Mg_0.2_ alloy. The optimal compositions for the Al-Mn-Zn-Mg-Si system present limited availability for solid solution formation structures. Only three out of seven compositions are placed well in the required intervals. Due to the targeted application that originated the research study, an alloy with density close to 3 g/cm^3^ was desired. The Al_3_Mn_0.2_Zn_0.3_Mg_0.7_Si_0.8_ has the lowest density and presents similar criteria values with the other low-density alloys.

### 3.2. CALPHAD Modeling

The CALPHAD method is using thermodynamic and kinetic data and equations to determine Gibbs free energies and diffusion mobility characteristics of a system [22]. Phase stability parameters and phase composition can be determined function of alloy composition and system temperature. Recent improvements of alloys databases allow for the obtaining of pertinent results when using CALPHAD based software.

**Figure 5:**
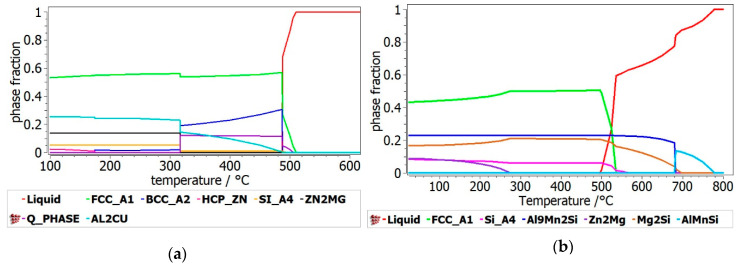
Phase composition versus temperature for the (**a**) Al_3.4_Cu_0.5_Si_0.2_Zn_0.5_Mg_0.2_ and (**b**) Al_3_Mn_0.2_Zn_0.3_Mg_0.7_Si_0.8_ alloys.

The resulted Al_3.4_Cu_0.5_Si_0.2_Zn_0.5_Mg_0.2_ phase diagram (Figure 5a) shows two different regions concerning the formation of hard intermetallic phases. Zn_2_Mg and Al_2_Cu based phases are in higher proportion bellow 316 °C and decrease significantly above this temperature. Presence of these compounds is determined by the high proportions of Zn, Mg, and Cu. At higher temperatures the The BCC-A2 and FCC-A1 disordered phases form at aprox. 488 °C through an invariant reaction from the liquid state. These structures were mentioned before in Cu and Si containing multicomponent alloys [33]. In the 316–488 °C range the alloy structure is composed mainly of disordered FFC-A1, BCC-A2 phases and multicomponent intermetallic Q-phase, which suggests that the alloy composition is favouring the complex structures at this temperature level. Modeling results show a near-equimolar composition of Al, Zn and Cu in the BCC-A2 phase and higher concentrations of Mg and Si in the Q-phase. The Al_2_Cu phase is also forming with a steady increase in concentration, in this temperature range. The BCC-A2 and Q-phase structures become unstable at approx. 316 °C participating in a second invariant transformation with the formation of stable Zn_2_Mg and Si-A4 phases. A substantial increase in the Al_2_Cu phase is also presented after this critical transformation. The ductile FCC-A1 phase is predominant all across the temperature range. For the Al_3_Mn_0.2_Zn_0.3_Mg_0.7_Si_0.8_ alloy (Figure 5b), two intermetallic phases of alpha-Al_9_Mn_2_Si and Mg_2_Si are shown to have great stability at lower and higher temperatures and form early during the solidification process. Same as in the previous alloy structure, the FCC_A1 phase is predominant at all temperatures, but starts forming after the intermetallic phases.

**Figure 6:**
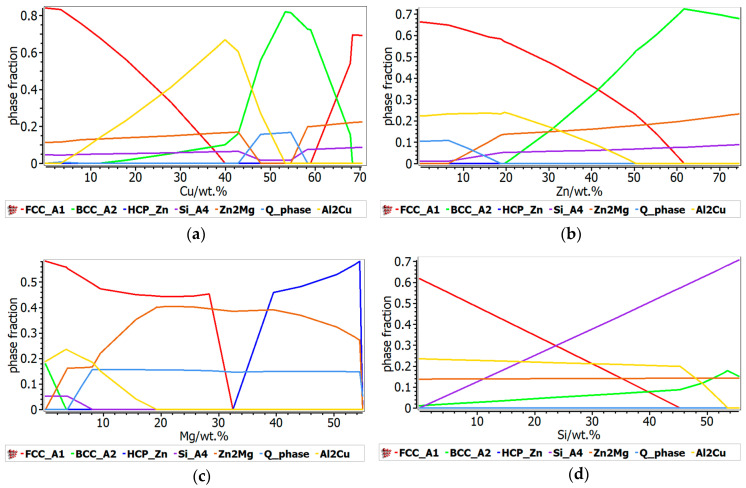
Influence of the element content over the phase stability in Al_3.4_Cu_0.5_Si_0.2_Zn_0.5_Mg_0.2_: (**a**) Cu influence; (**b**) Zn influence; (**c**) Mg influence; (**d**) Si influence.

The phase evolution function of element concentration was also calculated for both alloy systems. In the Al-Cu-Si-Zn-Mg system (Figure 6), Cu content is critical for the formation of the Al_2_Cu phase, with a peak at 40 wt.% were solid solutions are completely supressed. A predominant BCC solid solution structure is found when Cu concentration is between 50 and 60 wt.%. The main influence of Zn content is shown by the transition between the FCC and BCC at higher Zn concentrations. The total proportion of the intermetallic based phases remains constant with the increase of Zn content, with the transition between the formation of Al_2_Cu and Zn_2_Mg phases.

Higher concentrations of Mg have a strong influence over the formation of intermetallic based phases, mainly the Zn_2_Mg intermetallic compound, which becomes very stable over 8 wt.% Mg. Because most of the Mg is taken for the formation of Zn_2_Mg compound, the Si content does not influence the concentration of intermetallic based phases. The proportion of the FCC solid solution decreases with the increase of the Si content, which is typical for the Al-Si alloys.

**Figure 7:**
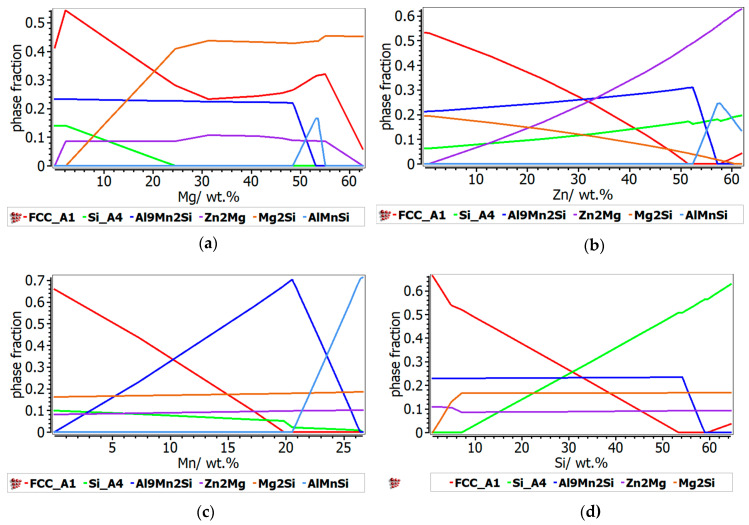
Influence of the element content over the phase stability in Al_3_Mn_0.2_Zn_0.3_Mg_0.7_Si_0.8_: (**a**) Mg influence; (**b**) Zn influence; (**c**) Mn influence; (**d**) Si influence.

The Al-Mn-Zn-Mg-Si alloy system (Figure 7) is mainly defined by the presence of Mn, which has an important contribution to the formation of complex compound-based phases. Thus, the increase of the Mn content induces a significant increase in the Al_9_Mn_2_Si type- complex compound phase, which reaches a peak of 0.7 phase fraction at just 20 wt.% Mn, after which decreases abruptly at the exchange of the AlMnSi phase. Meanwhile, the proportion of FCC and BCC phases suffer a major decrease, with under 0.1 phase fraction at 20 wt.% Mn. Zn influence on the phase stability is similar to the Al-Cu-Si-Zn-Mg system, with the difference consisting in the formation of Mg_2_Si phase instead of Al_2_Cu phase. At lower Zn concentrations, Mg_2_Si is more stable than Zn_2_Mg. The FCC-A1 phase is decreasing strongly at the exchange of the BCC phase formation and the Al_9_Mn_2_Si type phase is dominant at up to 55 wt.% Zn. As in the Al-Cu-Si-Zn-Mg system, the increase in Mg concentration determines a high increase on the intermetallic compounds proportion, thus the Mg_2_Si compound is predominant after 20 wt.% Mg. As expected, the influence of Si content in the phase evolution of Al-Mn-Zn-Mg-Si structure is very similar to the Al-Cu-Si-Zn-Mg system.

**Figure 8:**
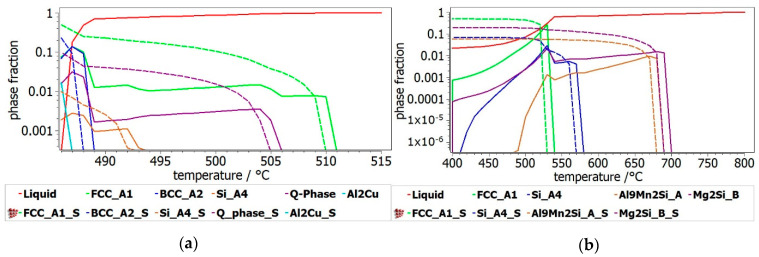
Scheil–Gulliver solidification diagrams for (**a**) Al_3.4_Cu_0.5_Si_0.2_Zn_0.5_Mg_0.2_ alloy and (**b**) Al_3_Mn_0.2_Zn_0.3_Mg_0.7_Si_0.8_ alloy.

**Figure 9:**
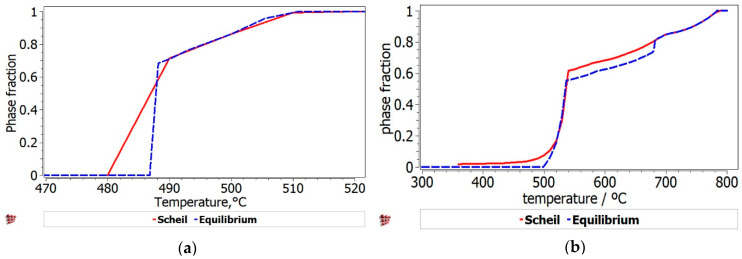
Equilibrium and Scheil–Gulliver liquidus solidification curves for the: (**a**) Al_3.4_Cu_0.5_Si_0.2_Zn_0.5_Mg_0.2_ alloy and (**b**) Al_3_Mn_0.2_Zn_0.3_Mg_0.7_Si_0.8_ alloy.

The phases formed during non-equilibrium solidification, modeled with Scheil–Gulliver method, are shown in Figure 8. The behavior of the phases with termination S describes the cumulative solidification patterns, containing equilibrium and non-equilibrium values. The equilibrium and non-equilibrium solidification are similar for the Al_3_Mn_0.2_Zn_0.3_Mg_0.7_Si_0.8_ alloy (Figure 8a), while in the Al_3.4_Cu_0.5_Si_0.2_Zn_0.5_Mg_0.2_ alloy (Figure 8b) small differences are revealed between solidification temperatures. Obviously, the Al_3.4_Cu_0.5_Si_0.2_Zn_0.5_Mg_0.2_ alloy presents a pattern characteristic to castable aluminum alloys, having also a smaller solidification temperature than Al_3_Mn_0.2_Zn_0.3_Mg_0.7_Si_0.8_ (Figure 9). FCC-A1 type solid solution solidifies first in the Al_3.4_Cu_0.5_Si_0.2_Zn_0.5_Mg_0.2_ alloy, while the solidification of Al_3_Mn_0.2_Zn_0.3_Mg_0.7_Si_0.8_ alloy is mostly defined by the behavior of Al_9_Mn_2_Si and Mg_2_Si intermetallic phases. The AlMnSi phase is unstable at lower temperatures and transforms into Al_9_Mn_2_Si at later stages.

### 3.3. Kinetics Simulation

**Figure 10:**
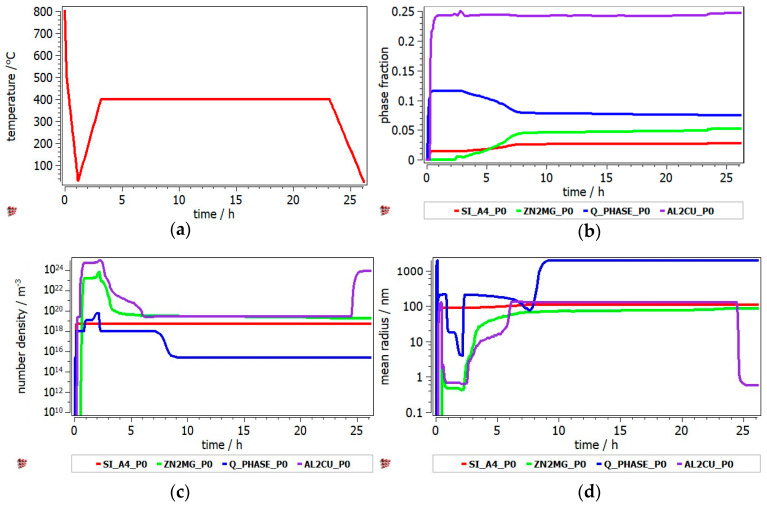
Precipitation kinetics in Al_3.4_Cu_0.5_Si_0.2_Zn_0.5_Mg_0.2_ alloy during annealing at 400 °C, for 2 h: (**a**) heat treatment diagram, (**b**) precipitate phase fraction versus time, (**c**) precipitate number density versus time, (**d**) precipitate mean radius versus time.

**Figure 11:**
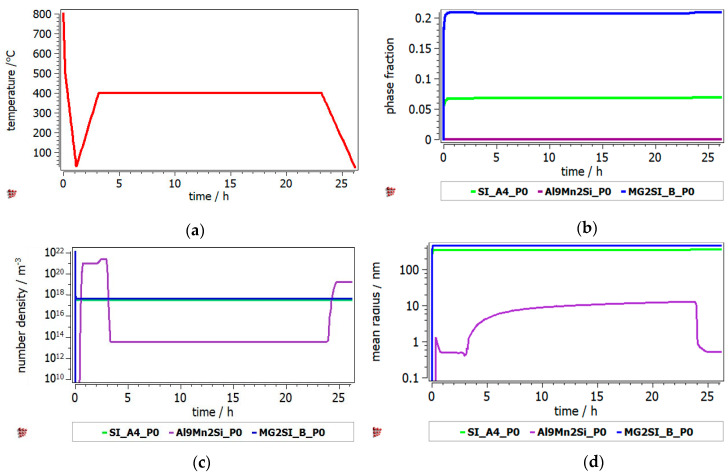
Precipitation kinetics in Al_3_Mn_0.2_Zn_0.3_Mg_0.7_Si_0.8_ alloy during annealing at 400 °C, for 2 h: (**a**) heat treatment diagram, (**b**) precipitate phase fraction versus time, (**c**) precipitate number density versus time, (**d**) precipitate mean radius versus time.

The heat treatment of Al_3.4_Cu_0.5_Si_0.2_Zn_0.5_Mg_0.2_ and Al_3_Mn_0.2_Zn_0.3_Mg_0.7_Si_0.8_ alloys was studied by the identification of hard phase precipitation in the FCC_A1 matrix. It can be seen from Figure 10 that the Al_2_Cu precipitate has the highest fraction, while the precipitate radius is small and uniform across the annealing interval. The Q-phase is also shown in the Al_3.4_Cu_0.5_Si_0.2_Zn_0.5_Mg_0.2_ precipitation diagram, but in lower proportion. The precipitation kinetics of the Al_3_Mn_0.2_Zn_0.3_Mg_0.7_Si_0.8_ alloy (Figure 11) shows a close competition between the Al_9_Mn_2_Si-type and Mg_2_Si compounds. Nevertheless, the size of the complex compound is significantly smaller than that of the Mg_2_Si. The Si_A4 phase is most likely found as eutectic in both alloys. Even if the AlMnSi precipitate is showing on the diagram, appears to be a nonequilibrium phase at 400 °C and is not stable during the heat treatment stage.

### 3.4. Experimental Results

The experimental chemical composition of the alloys is presented in Table 4. The resulted alloy specimens have each element composition within a maximum of 2 wt.% interval from the nominal values. Due to the high percentage of the elements, a variation of 2 wt.% in composition has little influence on the structural behavior of the alloy, and the analyses implied in the paper has the meaning in offering a mainly qualitative and not standardized information related to the studied alloys characteristics.

**Figure 12:**
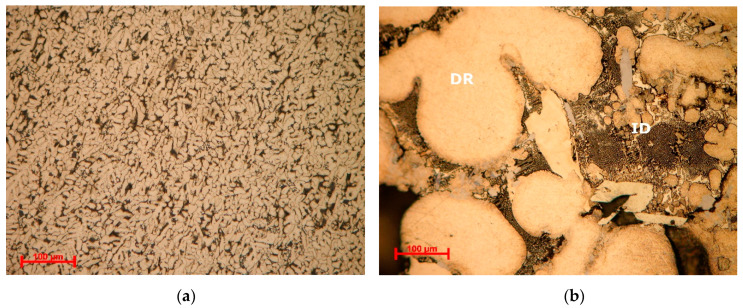
Optical micrographs of (**a**) as-cast and (**b**) annealed Al_3.4_Cu_0.5_Si_0.2_Zn_0.5_Mg_0.2_ alloy. DR- dendritic area, ID-interdendritic area.

**Figure 13:**
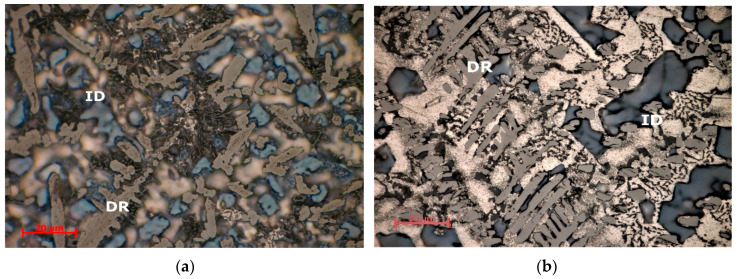
Optical micrographs of (**a**) as-cast and (**b**) annealed cast Al_3_Mn_0.2_Zn_0.3_Mg_0.7_Si_0.8_ alloy. DR—dendritic area; ID—interdendritic area.

Optical analyses of the alloy samples (Figure 12 and Figure 13) revealed significantly different morphologies between as cast and heat-treated states. Optical microscopy results for the as cast Al_3.4_Cu_0.5_Si_0.2_Zn_0.5_Mg_0.2_ alloys showed a fine and uniform dispersed dendritic structure (Figure 12a). Several phases are found in the interdendritic area, including occasional eutectic formations. The Al_3.4_Cu_0.5_Si_0.2_Zn_0.5_Mg_0.2_ heat treated alloy (Figure 12b) shows a dendritic structure with several micropores, mainly placed in the interdendritic area. In average, less than 5 vol % porosity was found in the specimens. The dendrite size is significantly larger than in the as-cast alloy, with well-defined interdendritic phases. Eutectic formations were also identified in the heat-treated sample. The as-cast and heat treated Al_3_Mn_0.2_Zn_0.3_Mg_0.7_Si_0.8_ samples (Figure 13a,b) show a similar fine dendritic structure.

Well defined, but small dendrite secondary arms are present in the structures. Two different types of dendritic formations are identified. Micropores are present throughout both samples. Microporosity level was determined as 3 vol % from the analysed samples. Etching and higher magnification of the as-cast and heat treated Al_3_Mn_0.2_Zn_0.3_Mg_0.7_Si_0.8_ alloy samples show a polyphasic interdendendritic structure, with significant interdendritic eutectic. Hard intermetallic phases are present at similar sizes in both samples.

**Figure 14:**
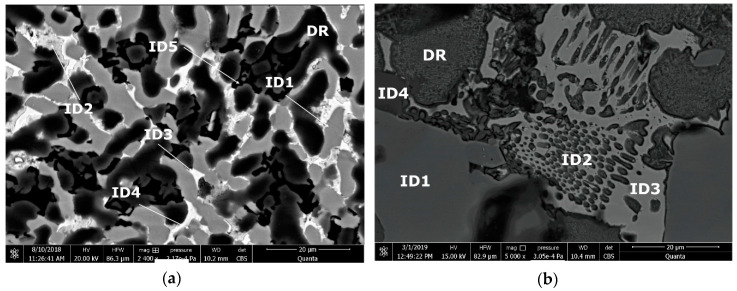
SEM images of (**a**) as-cast and (**b**) annealed cast Al_3.4_Cu_0.5_Si_0.2_Zn_0.5_Mg_0.2_ alloy. DR—dendritic area; ID—interdendritic area; ID1,2,3, and 4—interdendritic phases (Table 4).

**Figure 15:**
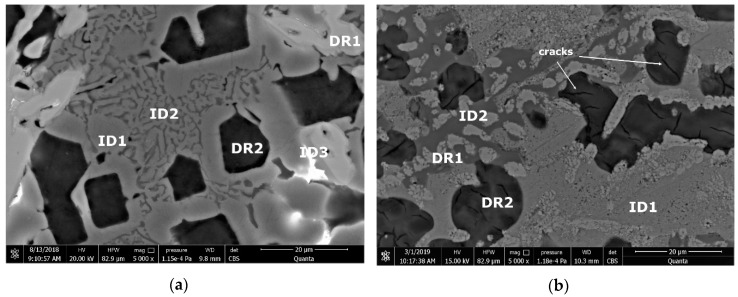
SEM images of (**a**) as-cast and (**b**) annealed cast Al_3_Mn_0.2_Zn_0.3_Mg_0.7_Si_0.8_ alloy. DR—dendritic area; ID—interdendritic area; ID1,2,3, and 4—interdendritic phases (Table 5).

**Figure 16:**
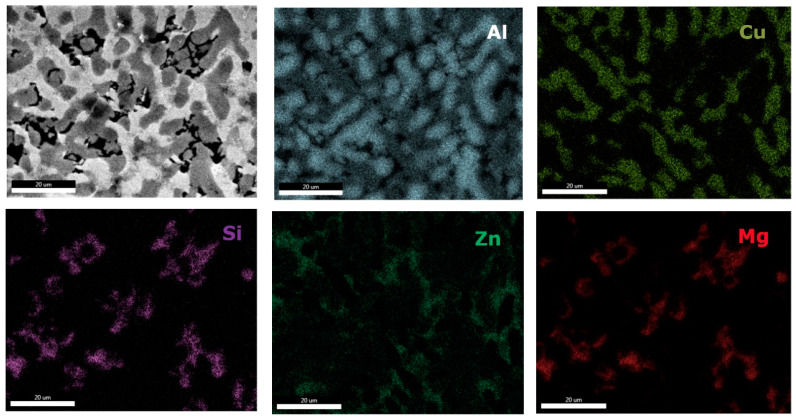
EDS mapping of as-cast Al_3.4_Cu_0.5_Si_0.2_Zn_0.5_Mg_0.2_ alloy.

**Figure 17:**
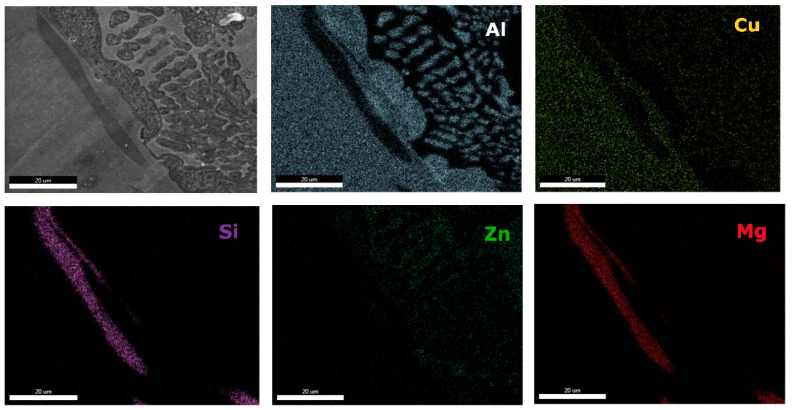
EDS mapping of heat treated Al_3.4_Cu_0.5_Si_0.2_Zn_0.5_Mg_0.2_ alloy.

Microstructural analyses through scanning electron microscopy (Figure 14 and Figure 15), shows large difference between the as-cast and heat-treated alloys. The as-cast Al_3.4_Cu_0.5_Si_0.2_Zn_0.5_Mg_0.2_ (Figure 14a) is formed of multiple phases of similar size and uniform distribution in the material. After heat treatment the alloy exhibits a large dendrite structure and well-defined interdendritic eutectic (Figure 14b). The composition of Al_3.4_Cu_0.5_Si_0.2_Zn_0.5_Mg_0.2_ phase structures, for as-cast and heat-treated states are presented in Table 5. Results for EDS analyses of Al_3.4_Cu_0.5_Si_0.2_Zn_0.5_Mg_0.2_ alloy reveals high concentration of Al and Zn in the dendritic area (DR). Cu is found mainly in the interdendritic area (ID), forming well defined phases with Al, Zn, and Mg (ID1, ID2, ID3, ID4, and ID5). The eutectic formation investigated with EDS-mapping (Figure 16 and Figure 17), is composed of Al and Zn based constituents. Mg and Si are found concentrated in common regions, most likely forming intermetallic compounds.

The as cast and heat-treated structures for Al_3_Mn_0.2_Zn_0.3_Mg_0.7_Si_0.8_ alloy (Figure 15) show similar phase sizes with small differences related to the distribution of interdendritic eutectic formations. Dark intermetallic phases with dendritic appearance are well distinguished at high magnifications, presenting small internal cracks in the heat-treated sample.

**Figure 18:**
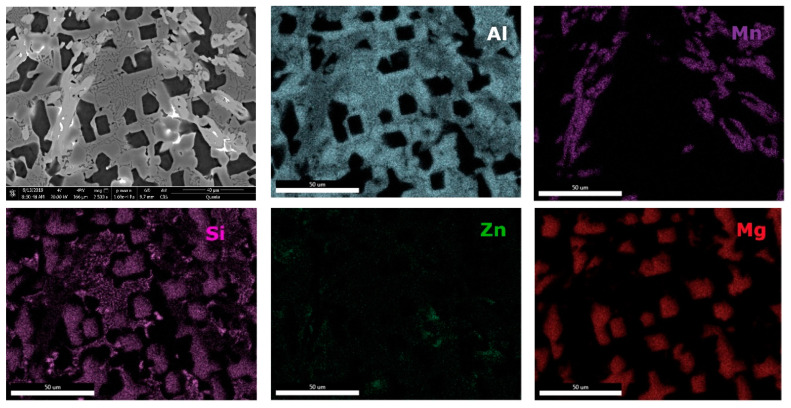
EDS mapping of as-cast Al_3_Mn_0.2_Zn_0.3_Mg_0.7_Si_0.8_ alloy.

**Figure 19:**
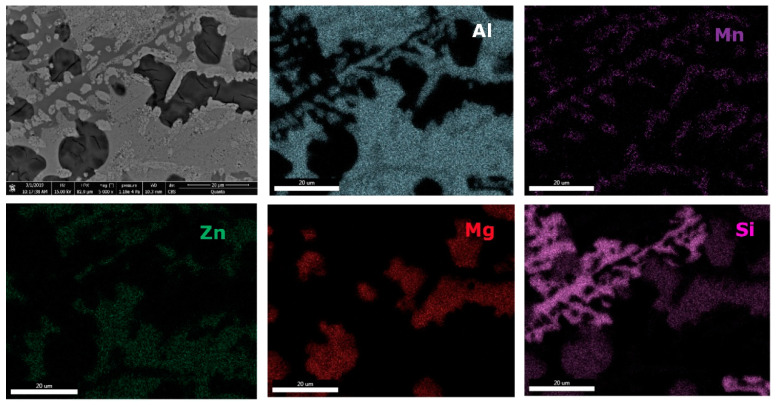
EDS mapping of heat treated Al_3_Mn_0.2_Zn_0.3_Mg_0.7_Si_0.8_ alloy.

The interdendritic area indicates differences in terms of phase size and distribution, both samples revealing multiple phases. Phase composition determined by EDS analysis (Table 6) showed Al-Mn and Mg-Si based dendritic formations. Three separate interdendritic phases, with various concentrations of the elements (ID1, ID2, and ID3), were distinguished in the as-cast sample. Al is present in larger quantity in the interdendritic area forming a continuous phase with Zn. An occasional phase composed of Al, Mn, Si, and Zn was also identified in the as-cast sample. Si was also identified segregated in the interdendritic area. Similar compositions were found in the heat-treated sample, setting aside a minor phase with large Zn concentration. The EDS-mapping results (Figure 18 and Figure 19) confirm the previous findings. The well-dispersed hard phases are composed mostly of Si and Mg. Si segregations appear as long branches in certain regions. Al and Mn are found highly concentrated in the dendritic area.

**Figure 20:**
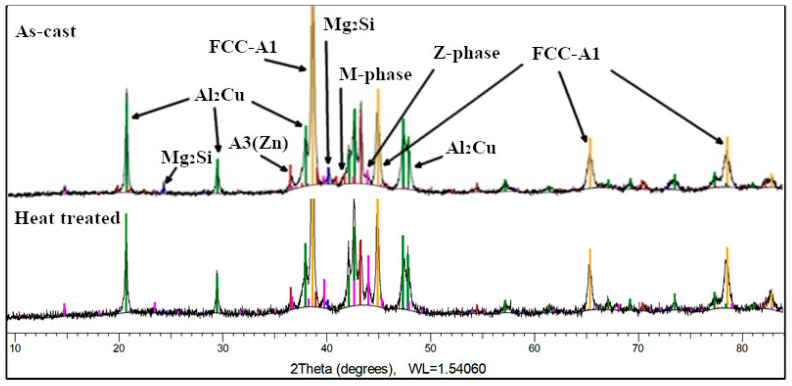
XRD results for as-cast and heat treated Al_3.4_Cu_0.5_Si_0.2_Zn_0.5_Mg_0.2_ alloy.

The X-ray analysis of the as-cast Al_3.4_Cu_0.5_Si_0.2_Zn_0.5_Mg_0.2_ alloy (Figure 20) indicates a structure composed of Al and Zn based solid solutions and Al_2_Cu, Mg_2_Zn_11_, Mg_2_Si, MgZn_2_ intermetallic compound-based phases. Phase proportion calculations revealed a high content of FCC-A1 (Al) solid solution (48 wt.%) and Al_2_Cu-C16 phase (34 wt.%). The HCP-A3 (Zn) solid solution (8 wt.%), Z phase of type (Cu_6_Mg_2_Al_5_) Mg_2_Zn_11_ (4 wt.%), M phase of type (CuMgAl)MgZn_2_ (4 wt.%) and MgSi_2_ (2 wt.%) have minority proportions. After the heat treatment performed at 400 °C for 20 h, it can be noted that the hexagonal phase M (a quaternary continuous solid solution, formed between the ternary compound CuMgAl and the binary compound MgZn_2_) is no longer present in the alloy structure and the cubic phase Z (a quaternary continuous solid solution, between the ternary compound Cu_6_Mg_2_Al_5_ and the binary compound Mg_2_Zn_11_) increases in concentration. In the heat-treated sample, small percentage changes of the FCC-A1 type (50 wt.%) and Al_2_Cu-C16 type (31 wt.%) phases are also observed, while Mg_2_Si decreases significantly (1.7 wt.%).

**Figure 21:**
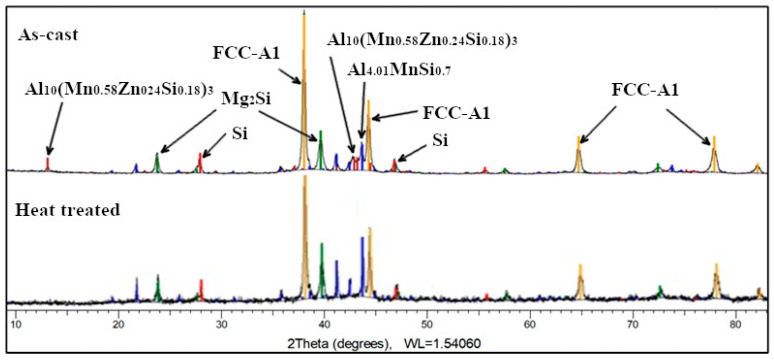
XRD results for as-cast and heat treated Al_3_Mn_0.2_Zn_0.3_Mg_0.7_Si_0.8_ alloy.

The as-cast Al_3_Mn_0.2_Zn_0.3_Mg_0.7_Si_0.8_ alloy structure (Figure 21) consists of solid solutions and intermetallic compound phases. The FCC-Al (Al) type solid solution is predominant (55 wt.%), while Al_4.01_MnSi_0.74_ (16 wt.%), Al_10_(Mn_0.58_Zn_0.24_Si_0.18_)_3_ (10 wt.%), and Mg_2_Si (13 wt.%) intermetallics are in much less proportions. Si was found also at 6 wt.%. The Al_10_(Mn_0.58_Zn_0.24_Si_0.18_)_3_ is a hexagonal complex phase formed from the binary compound Al_10_Mn_3_ and the ternary compound (Al_9_Si) Mn_3_. The diffraction spectrum for the heat treated Al_3_Mn_0.2_Zn_0.3_Mg_0.7_Si_0.8_ alloy indicates a four-phase composition. The Al_10_(Mn_0.58_Zn_0.24_Si_0.18_)_3_ phase is no longer present which contributes to the increase of Al_4.01_MnSi_0.74_ (32 wt.%) and Mg_2_Si (16 wt.%) phases, at the expense of FCC-A1 phase (46 wt.%).

**Figure 22:**
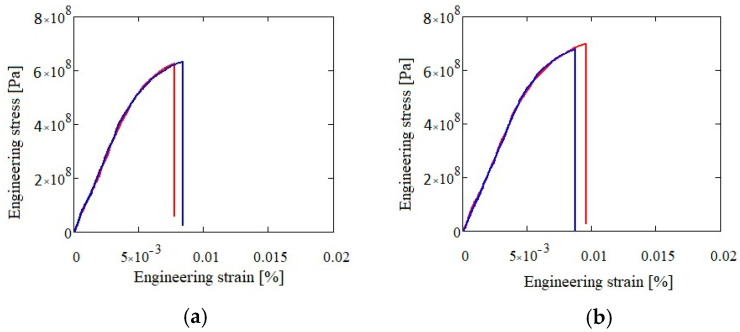
Compression test results for as-cast (**a**) and heat treated (**b**) Al_3.4_Cu_0.5_Si_0.2_Zn_0.5_Mg_0.2_ alloy.

**Figure 23:**
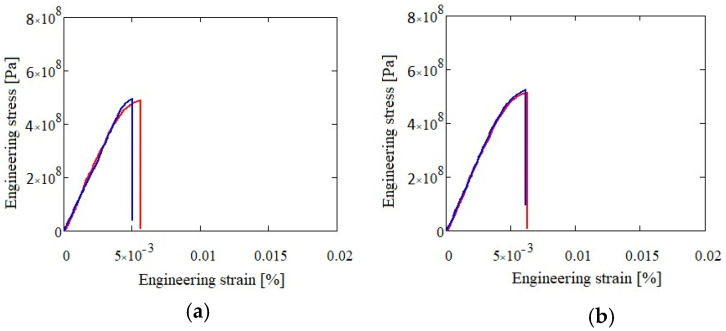
Compression test results for as-cast (**a**) and heat treated (**b**) Al_3_Mn_0.2_Zn_0.3_Mg_0.7_Si_0.8_ alloy.

The mechanical testing results for both alloys in as-cast and heat treated states are presented in Table 7, Figure 22 and Figure 23. The Al_3.4_Cu_0.5_Si_0.2_Zn_0.5_Mg_0.2_ alloy presented a slightly higher resistance (590 MPa in as-cast and 618 MPa in heat treated state) than the Al_3_Mn_0.2_Zn_0.3_Mg_0.7_Si_0.8_ alloy (486 MPa in as-cast and 507 MPa in heat treated state).

Both alloys showed a brittle behavior with elongation under 3%. The microhardness tests showed high values in comparison with the conventional aluminum alloys (A357.0 and A5083). The as-cast samples presented a fine and well distributed phase structure, which allowed for multiple determinations with similar values, averaged at 268 HV for Al_3.4_Cu_0.5_Si_0.2_Zn_0.5_Mg_0.2_ alloy and at 277 HV for the Al_3_Mn_0.2_Zn_0.3_Mg_0.7_Si_0.8_ alloy. However, the annealed structures presented large and well-defined phases, so microhardness indentations revealed individual values for each distinct phase. The rounded proportional average of the determined values for the annealed alloys were: 274 HV for Al_3.4_Cu_0.5_Si_0.2_Zn_0.5_Mg_0.2_ and 283 HV for Al_3_Mn_0.2_Zn_0.3_Mg_0.7_Si_0.8_. The results showed similar microhardness values between the two alloy states.

## 4. Discussion

The thermodynamic and Hume-Rothery rules for the formation of solid solution structures offer a good estimate for the selection of most appropriate compositions, but cannot fully predict real life experimental results. The targeted alloys were intentionally annealed to eliminate inherent out of equilibrium structures that may appear after the casting process.

Selection criteria applied to the Al_3.4_Cu_0.5_Si_0.2_Zn_0.5_Mg_0.2_ alloy showed optimal values for most of the parameters. The electronegativity difference was the only parameter that was above the recommended limit. By comparison, the selected Al_3_Mn_0.2_Zn_0.3_Mg_0.7_Si_0.8_ alloy presented most of the criteria parameters out of the recommended limits, which suggests a structure with a higher percentage of intermetallic compounds. For both alloys, the experimental findings matched the criteria calculations and showed a high percentage of the FCC-Al phase and presence of intermetallic compounds. For the Al_3_Mn_0.2_Zn_0.3_Mg_0.7_Si_0.8_ alloy was found a significantly larger number of intermetallic phases.

CALPHAD method produced similar phase configurations with those obtained in the experimental results, with slight changes for low proportion phases. Thus, Si suggested by MatCalc was not found in the Al_3.4_Cu_0.5_Si_0.2_Zn_0.5_Mg_0.2_ heat treated sample, instead Mg_2_Zn_11_ was detected at X-ray analysis. The Q-phase was determined as a stable phase in the studied alloy and was not indicated by the analyses of the experimental samples. Thermodynamic calculations showed presence of Zn_2_Mg which were not detected in the Al_3_Mn_0.2_Zn_0.3_Mg_0.7_Si_0.8_ heat treated sample. The Al_9_Mn_2_Si phase shown in MatCalc simulation is very similar to the experimental Al_4.01_MnSi_0.74_. It is known that the stoichiometry of the Al_4.01_MnSi_0.74_ phase is similar to the stoichiometry of the α-Al_9_Mn_2_Si phase, thus indicating a similar crystal structure [34]. The proportion of phases was also well represented by the CALPHAD method.

There is also a good representation of the solidification behavior of the selected alloys obtained with the Scheil–Gulliver method. The resulted diagram for Al_3.4_Cu_0.5_Si_0.2_Zn_0.5_Mg_0.2_ alloy (Figure 8a) showed that the first phase to form is FCC-A1, which is indicated as a dendrite formation in the optical and SEM results. The Al_3_Mn_0.2_Zn_0.3_Mg_0.7_Si_0.8_ alloy has a more complex structure with two dendrite formations (Mg_2_Si and Al_9_Mn_2_Si), which are also indicated by the solidification simulation diagram (Figure 8b).

Precipitation kinetics was applied to determine the structural changes in the as-cast structure over the annealing process. MatCalc software was setup to predict the nucleation and growth of the precipitated intermetallic phases during the heat treatment process, starting with the as-cast structure. The simulation results for Al_3.4_Cu_0.5_Si_0.2_Zn_0.5_Mg_0.2_ alloy indicated a high proportion of the Al_2_Cu precipitate, which was maintained at the same value from the as-cast structure. The results were verified by X-ray analyses. The number density of Al_2_Cu decreases from as-cast to heat treated state, which remains consistent with the increase in the precipitate size, shown both by the simulation and SEM results. However, the size of the precipitate is much larger in the experimental specimens. The simulation results for the Al_3_Mn_0.2_Zn_0.3_Mg_0.7_Si_0.8_ shows important discrepancies against the experimental findings. The Mg_2_Si precipitate is in much larger proportion than the Al_9_Mn_2_Si precipitate in the MatCalc simulation, which is in contradiction with the X-ray analyses results. Still, there are similarities regarding the high stability of the Mg_2_Si precipitate number density and size, which can be observed from the simulation and microstructural characterization images.

The SEM-EDS and XRD results show a larger number of phases for the as-cast specimens. The complex M phase in Al_3.4_Cu_0.5_Si_0.2_Zn_0.5_Mg_0.2_ and complex phase Al_10_(Mn_0.58_Zn_0.24_Si_0.18_)_3_ in Al_3_Mn_0.2_Zn_0.3_Mg_0.7_Si_0.8_ are no longer present in the annealed state. This illustrates the metastable structure that is present in the as-cast samples and that slow diffusion processes are taking place in complex concentrated alloys. There is also a large difference between the behavior of the selected alloys during the annealing process. The Al_3.4_Cu_0.5_Si_0.2_Zn_0.5_Mg_0.2_ alloy suffers large transformations in phase size and distribution, similar to the castable aluminum alloys, while the Al_3_Mn_0.2_Zn_0.3_Mg_0.7_Si_0.8_ presents small changes in structural configuration, similar to the wrought aluminum alloys.

The mechanical properties of the alloys showed relatively high values for compression yield strength and hardness, in comparison with conventional aluminum alloys. This was expected due to the higher content of intermetallic phases. Unfortunately, the stress strain curves showed a brittle behavior that is detrimental for practical use. Even if the structural change is significant after annealing, with a significantly larger grain size, the Al_3.4_Cu_0.5_Si_0.2_Zn_0.5_Mg_0.2_ retains similar values for the mechanical properties. This aspect could be analysed further in future studies. The Al_3_Mn_0.2_Zn_0.3_Mg_0.7_Si_0.8_ alloy presents no significant changes in grain size after annealing (typical to wrought alloys) and consequently the mechanical properties present similar values for both alloy states.

Overall, the tools used for the prediction of the lightweight CCAs structures were in good agreement with the experimental findings. Small differences shown between simulation and experimental results indicate inherent errors that may appear when calculating multicomponent alloys with a large proportion of elements. Elemental diffusion plays an important role in the evolution of the alloys structure, leading to the formation of complex phases, which are hard to be determined by conventional analysis methods and also difficult to be simulated by present software.

The selected compositions show a good potential for obtaining practical, low cost LWCCA, with improved properties. The substantial increase in the alloy’s elements composition does not show dramatical changes in the structural behaviors. The intermetallic phases induced by the higher composition of the reactive elements, typical for LWCCA, are not necessarily detrimental to the final properties. A uniform distribution and reduced size of the precipitates can lead to a stable structure with high mechanical properties at higher operation temperatures. However, future studies are recommended for further compositional tuning and customised heat treatment processes to obtain optimal configurations for targeted applications.

## 5. Conclusions

The selection and analyses of low weight complex concentrated alloys, with common and less expensive elements, before and after heat treatment, were presented in the paper. The selection of alloys with low density and high solid solution content from Al-Cu-Si-Zn-Mg and Al-Mn-Zn-Mg-Si systems was achieved by the optimization of previously established semiempirical criteria. Results showed that a high aluminum content determined better solid solution forming ability for both alloy systems. While Cu, Zn, and Mn have a beneficial influence on the criteria parameters, the increase in element content affects the density of the alloy. A balance between density and criteria optimal values, modeled with an optimization software, was applied to offer practical solutions in the selection process.

The selected Al_3.4_Cu_0.5_Si_0.2_Zn_0.5_Mg_0.2_ and Al_3_Mn_0.2_Zn_0.3_Mg_0.7_Si_0.8_ alloys were analysed for structural behavior by CALPHAD, solidification and kinetics simulations. Phase diagrams showed the formation of a multiphase structure with preponderant solid solutions. Intermetallic compound-based phases were found at lower concentrations but still significantly higher than in the conventional aluminum alloys. Two invariant transformations were indicated for Al_3.4_Cu_0.5_Si_0.2_Zn_0.5_Mg_0.2_ alloy, with a BCC-A2 phase stable between 316–488 °C. The influence of the elements on the structural evolution were similar with the results obtained from semiempirical criteria calculations. The non-equilibrium solidification simulation discussed the order of which the phases are solidifying in normal casting conditions. The soft FCC phase is stabilizing first in the Al_3.4_Cu_0.5_Si_0.2_Zn_0.5_Mg_0.2_ alloy, while intermetallic compound phases Mg_2_Si and Al_9_Mn_2_Si are forming first in the Al_3_Mn_0.2_Zn_0.3_Mg_0.7_Si_0.8_ alloy. The diffusion driven precipitation simulation offered indications on the formation of intermetallic precipitates in the solid solution matrix, showing that the Al_2_Cu and Mg_2_Si phases will be stabilizing at highest concentration levels for the Al_3.4_Cu_0.5_Si_0.2_Zn_0.5_Mg_0.2_ and Al_3_Mn_0.2_Zn_0.3_Mg_0.7_Si_0.8_ alloys, respectively.

The experimental findings, provided by optical, SEM-EDS, and XRD analyses, indicated that both alloys presented complex structures, containing mostly solid solutions, but also a significant amount of intermetallic phases. The Al_3.4_Cu_0.5_Si_0.2_Zn_0.5_Mg_0.2_ presented a refined structure in the as-cast state with a predominant Al solid solution phase. The Al_2_Cu and Mg-Zn intermetallics were also present in the alloy structure and continued to remain at a high percentage in the heat-treated samples. A dendritic structure based on FCC-Al phase was indicated in the Al_3.4_Cu_0.5_Si_0.2_Zn_0.5_Mg_0.2_ alloy. The Al_3_Mn_0.2_Zn_0.3_Mg_0.7_Si_0.8_ alloy showed a refined structure that was maintained also after the annealing process. This time the dendritic formations were indicated to be intermetallic compound phases: Mg_2_Si and Al_4.01_MnSi_0.74_. Between the as-cast and heat-treated state, a transformation of less stable Al_10_(Mn_0.58_Zn_0.24_Si_0.18_)_3_ into Al_4.01_MnSi_0.74_ was revealed, which lead to a significant increase in the concentration of the Al_4.01_MnSi_0.74_ compound (32 wt.%). The mechanical characterisation of the alloys revealed a high compression strength for both alloys (aprox 600 Mpa for Al_3.4_Cu_0.5_Si_0.2_Zn_0.5_Mg_0.2_ and aprox. 500 MPa for Al_3_Mn_0.2_Zn_0.3_Mg_0.7_Si_0.8_) and brittle behavior. The microhardness was found in the range of hard aluminum alloys (250–300 HV).

The comparison of the experimental results with the criteria and simulation results showed good representations. There were small differences in phase composition and precipitation behavior between the CALPHAD equilibrium diagrams for both alloys, kinetics simulation, and experimental results for the Al_3_Mn_0.2_Zn_0.3_Mg_0.7_Si_0.8_ alloy. These relative inconsistencies could be resolved in the future development of the software by improving the alloys database and the diffusion simulation, which are difficult to achieve for multicomponent alloys with reactive elements.

The intention of this study was to provide a path in the selection of multicomponent CCAs, containing less expensive raw materials, for lightweight applications, using tools that are readily available. The optimization criteria can be easily modified to fit required properties. The alloying examples presented in the paper were studied from several angles to give an indication of the expected structural behavior in this type of alloys.

## Figures and Tables

**Table 1:** Criteria calculation results for AlCuSiZnMg and AlMnZnMgSi systems.

Nr	Composition							∆χ_Allen_ %	Ω	Λ (J/mol·K)	ρ (g/cm^3^)
	Alloy	at %									
		Al	Cu	Si	Zn	Mg	Mn				
1	Al_0.5_CuSiZnMg	11.2	22.2	22.2	22.2	22.2	-	14.08	1.00	0.10	3.48
2	AlCuSiZnMg	20	20	20	20	20	-	13.42	1.09	0.11	3.39
3	Al_1.5_CuSiZnMg	27.3	18.2	18.2	18.2	18.2	-	12.85	1.15	0.12	3.32
4	Al_2_CuSiZnMg	33.3	16.7	16.7	16.7	16.7	-	12.34	1.19	0.13	3.26
5	Al_2.5_CuSiZnMg	38.5	15.4	15.4	15.4	15.4	-	11.89	1.23	0.13	3.21
6	AlCuSi_0.5_ZnMg	22.2	22.2	11.1	22.2	22.2	-	13.21	1.52	0.13	3.56
7	AlCuSi_1.5_ZnMg	18.2	18.2	27.3	18.2	18.2	-	13.41	0.93	0.10	3.26
8	AlCuSi_2_ZnMg	16.7	16.7	33.3	16.7	16.7	-	13.28	0.85	0.09	3.16
9	AlCuSi_2.5_ZnMg	15.4	15.4	38.5	15.4	15.4	-	13.09	0.81	0.09	3.08
10	AlCuSiZn_0.5_Mg	22.2	22.2	22.2	11.1	22.2	-	14.03	1.00	0.10	3.35
11	AlCuSiZn_1.5_Mg	18.2	18.2	18.2	27.3	18.2	-	12.88	1.16	0.12	3.43
12	AlCuSiZn_2_Mg	16.7	16.7	16.7	33.3	16.7	-	12.41	1.21	0.13	3.46
13	AlCuSiZn_2.5_Mg	15.4	15.4	15.4	38.5	15.4	-	11.98	1.25	0.14	3.50
14	AlCuSiZnMg_0.5_	22.2	22.2	22.2	22.2	11.1	-	11.60	1.10	0.13	3.70
15	AlCuSiZnMg_1.5_	18.2	18.2	18.2	18.2	27.3	-	14.55	1.07	0.10	3.16
16	AlCuSiZnMg_2_	16.7	16.7	16.7	16.7	33.3	-	15.27	1.06	0.10	2.99
17	AlCuSiZnMg_2.5_	15.4	15.4	15.4	15.4	38.5	-	15.74	1.04	0.10	2.86
18	AlCu_0.5_SiZnMg	22.2	11.1	22.2	22.2	22.2	-	13.68	0.97	0.10	2.90
19	AlCu_1.5_SiZnMg	18.2	27.3	18.2	18.2	18.2	-	13.11	1.18	0.12	3.80
20	AlCu_2_SiZnMg	16.7	33.3	16.7	16.7	16.7	-	12.79	1.26	0.12	4.16
21	AlCu_2.5_SiZnMg	15.4	38.5	15.4	15.4	15.4	-	12.47	1.32	0.12	4.47
22	Al_0.5_MnZnMgSi	11.1	-	22.2	22.2	22.2	22.2	13.34	0.60	0.11	3.92
23	AlMnZnMgSi	20	-	20	20	20	20	12.68	0.64	0.12	3.80
24	Al_1.5_MnZnMgSi	27.3	-	18.2	18.2	18.2	18.2	12.11	0.66	0.13	3.71
25	Al_2_MnZnMgSi	33.3	-	16.7	16.7	16.7	16.7	11.61	0.68	0.14	3.63
26	Al_2.5_MnZnMgSi	38.5	-	15.4	15.4	15.4	15.4	11.17	0.70	0.15	3.56
27	AlMn_0.5_ZnMgSi	22.2	-	22.2	22.2	22.2	11.1	13.22	0.73	0.11	3.53
28	AlMn_1.5_ZnMgSi	18.2	-	18.2	18.2	18.2	27.3	12.19	0.58	0.13	4.04
29	AlMn_2_ZnMgSi	16.7	-	16.7	16.7	16.7	33.3	11.74	0.55	0.14	4.25
30	AlMn_2.5_ZnMgSi	15.4	-	15.4	15.4	15.4	38.5	11.34	0.53	0.15	4.44
31	AlMnZn_0.5_MgSi	22.2	-	11.1	22.2	22.2	22.2	13.30	0.57	0.11	3.48
32	AlMnZn_1.5_MgSi	18.2	-	27.3	18.2	18.2	18.2	12.14	0.69	0.14	4.07
33	AlMnZn_2_MgSi	16.7	-	33.3	16.7	16.7	16.7	11.66	0.73	0.14	4.30
34	AlMnZn_2.5_MgSi	15.4	-	38.5	15.4	15.4	15.4	11.23	0.77	0.15	4.49
35	AlMnZnMg_0.5_Si	22.2	-	22.2	11.1	22.2	22.2	10.91	0.55	0.14	4.12
36	AlMnZnMg_1.5_Si	18.2	-	18.2	27.3	18.2	18.2	13.76	0.70	0.12	3.56
37	AlMnZnMg_2_Si	16.7	-	16.7	33.3	16.7	16.7	14.46	0.76	0.11	3.37
38	AlMnZnMg_2.5_Si	15.4	-	15.4	38.5	15.4	15.4	14.91	0.81	0.11	3.21
39	AlMnZnMgSi_0.5_	22.2	-	22.2	22.2	11.1	22.2	12.11	0.78	0.16	3.99
40	AlMnZnMgSi_1.5_	18.2	-	18.2	18.2	27.3	18.2	12.88	0.56	0.11	3.65
41	AlMnZnMgSi_2_	16.7	-	16.7	16.7	33.3	16.7	12.90	0.52	0.10	3.53
42	AlMnZnMgSi_2.5_	15.4	-	15.4	15.4	38.5	15.4	12.81	0.49	0.09	3.43

**Table 2:** Multiobjective optimization results for Al-Cu-Si-Zn-Mg system.

Nr	Optimized Compositions						δ %	∆χ_Allen_ %	Ω	Λ (J/mol·K)	ρ (g/cm^3^)
	Alloy	at %									
		Al	Cu	Si	Zn	Mg					
1	Al_3.4_Cu_0.5_Si_0.2_Zn_0.5_Mg_0.2_	70.8	10.4	4.2	10.4	4.2	5.71	7.17	2.37	0.30	3.18
2	Al_3.4_Cu_0.5_Si_0.2_ZnMg_0.2_	64.2	9.4	3.8	18.9	3.8	5.45	6.89	2.82	0.35	3.22
3	Al_3.4_Cu_0.5_Si_0.2_Zn_1.5_Mg_0.2_	58.6	8.6	3.4	25.9	3.4	5.22	6.63	3.20	0.39	3.26
4	Al_4_Cu_0.15_Si_0.1_Zn_0.7_Mg_0.3_	77.7	2.9	1.9	13.6	3.9	4.04	5.58	5.86	0.41	3.35
5	Al_3_Cu_0.2_Si_0.1_Zn_0.7_Mg_0.2_	71.4	4.8	2.4	16.7	4.8	4.51	6.10	4.88	0.38	3.57
6	Al_3_Cu_0.3_Si_0.1_Zn_0.6_Mg_0.3_	69.8	7	2.3	14	7	5.05	6.93	3.91	0.32	3.49
7	Al_3_Cu_0.2_Si_0.1_Zn_0.8_Mg_0.2_	69.8	4.7	2.3	18.6	4.7	4.29	5.94	5.43	0.38	3.41

**Table 3:** Multiobjective optimization results for Al-Mn-Zn-Mg-Si system.

Nr	Optimized Compositions						δ %	∆χ_Allen_ %	Ω	Λ (J/mol·K)	ρ (g/cm^3^)
	Alloy	at %									
		Al	Mn	Si	Zn	Mg					
1	Al_3_Mn_0.2_Zn_0.5_Mg_0.5_Si_0.5_	63.8	4.3	10.6	10.6	10.6	7.55	9.14	0.37	0.17	3.08
2	Al_3_Mn_0.3_Zn_0.2_Mg_0.6_Si_0.6_	63.8	6.4	12.8	4.3	12.8	8.05	9.54	0.37	0.15	2.97
3	Al_3_Mn_0.2_Zn_0.3_Mg_0.6_Si_0.7_	62.5	4.2	14.6	6.3	12.5	8.59	10.19	0.37	0.13	2.86
4	Al_3_Mn_0.2_Zn_0.3_Mg_0.7_Si_0.8_	60	4	16	6	14	9.02	10.71	0.38	0.12	2.82
5	Al_3.5_Mn_0.2_Zn_0.7_Mg_0.2_Si_0.1_	74.5	4.3	2.1	14.9	4.3	4.09	5.67	6.06	0.41	3.39
6	Al_4_Mn_0.2_Zn_0.8_Mg_0.2_Si_0.1_	75.5	3.8	1.9	15.1	3.8	4.29	5.94	5.43	0.38	3.41
7	Al_4_ Mn_0.26_Zn_0.9_Mg_0.2_Si_0.1_	73.3	4.8	1.8	16.5	3.7	4.51	6.1	4.88	0.38	3.57

**Table 4:** Alloys chemical composition in weight percent.

Alloy	Type	Al	Mg	Si	Zn	Cu	Mn
Al_3.4_Cu_0.5_Si_0.2_Zn_0.5_Mg_0.2_	nominal	55	2.9	3.4	19.6	19	-
	actual	53.6	3.2	4.2	20.9	17.8	-
Al_3_Mn_0.2_Zn_0.3_Mg_0.7_Si_0.8_	nominal	53.6	11.3	14.8	13	-	7.2
	actual	53.4	10.8	15.5	11.6	-	8.4

**Table 5:** EDS analyses results for Al_3.4_Cu_0.5_Si_0.2_Zn_0.5_Mg_0.2_ alloy.

State	Phase	Composition, at %				
		Al	Cu	Si	Zn	Mg
as-cast	DR	90.22	2.32	-	7.46	-
	ID1	66.34	30.70	-	2.96	-
	ID2	48.55	3.68	-	44.55	3.22
	ID3	18.60	6.08		47.11	28.21
	ID4	2.74	0.71	32.23	2.06	62.26
	ID5	55.26	29.34	-	8.43	6.97
annealed	DR	89.45	-	-	10.55	-
	ID1	74.69	25.31	-	-	-
	ID2	22.29	8.96	-	66.34	2.41
	ID3	12.56	11.46	-	59.63	16.35
	ID4	4.23	8.54	30.15	2.62	54.46

**Table 6:** EDS analyses results for Al_3_Mn_0.2_Zn_0.3_Mg_0.7_Si_0.8_ alloy.

State	Phase	Composition, at %				
		Al	Mn	Si	Zn	Mg
as-cast	DR1	63.30	22.25	12.09	2.36	-
	DR2	6.87	4.88	29.78	1.30	57.17
	ID1	90.84	-	1.46	6.10	1.60
	ID2	20.61	-	75.31	4.08	-
	ID3	55.04	6.77	4.56	29.92	3.71
annealed	DR1	67.56	20.09	12.07	0.28	-
	DR2	1.85	-	37.54	0.89	59.72
	ID1	89.59	-	0.45	8.99	0.97
	ID2	8.23	5.12	83.01	2.73	0.91

**Table 7:** Alloys chemical composition in weight percent.

Alloy	State	Compression Yield Strength, (MPa)	Hardness, (HV)
Al_3.4_Cu_0.5_Si_0.2_Zn_0.5_Mg_0.2_	as-cast	590	268
	annealed	618	274
Al_3_Mn_0.2_Zn_0.3_Mg_0.7_Si_0.8_	as-cast	486	277
	annealed	507	283
A357.0	as-cast	240	120
AA5083	wrought	380	80
